# Supplementary figures and images for: A novel positive selection system for plant transformation based on microbial biuret hydrolase and biuret
Source: PLoS One. 2026 May 8;21(5):e0347957. doi: 10.1371/journal.pone.0347957 (PMC13155557; doi:10.1371/journal.pone.0347957)

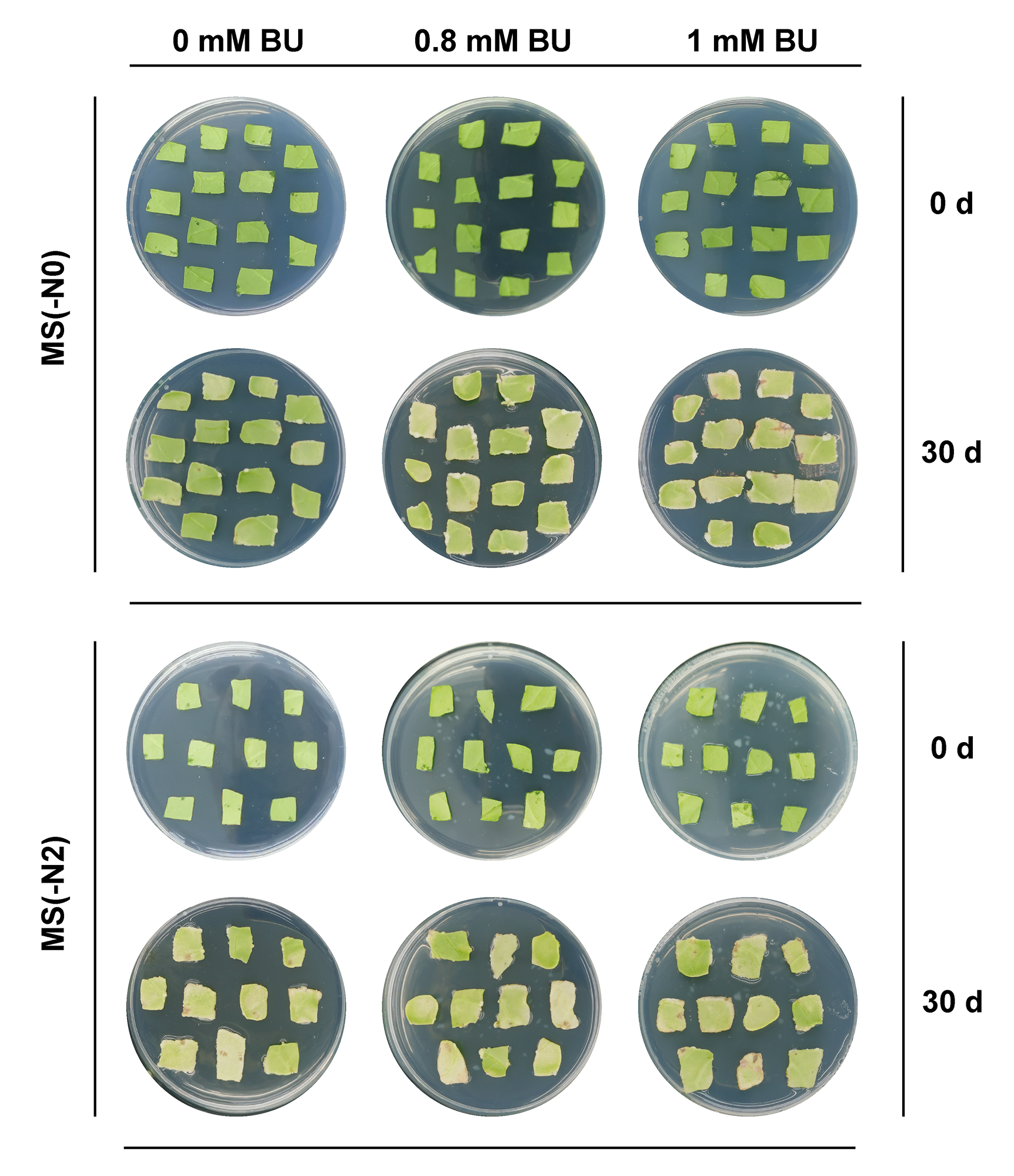

Supplement: S1 Fig — Explants were cultured for 30 days on media with varying nitrogen compositions: standard MS(-N0) and MS(-N2), each supplemented with a gradient of BU concentrations (0–1 mM). (TIF) [file pone.0347957.s001.tif]

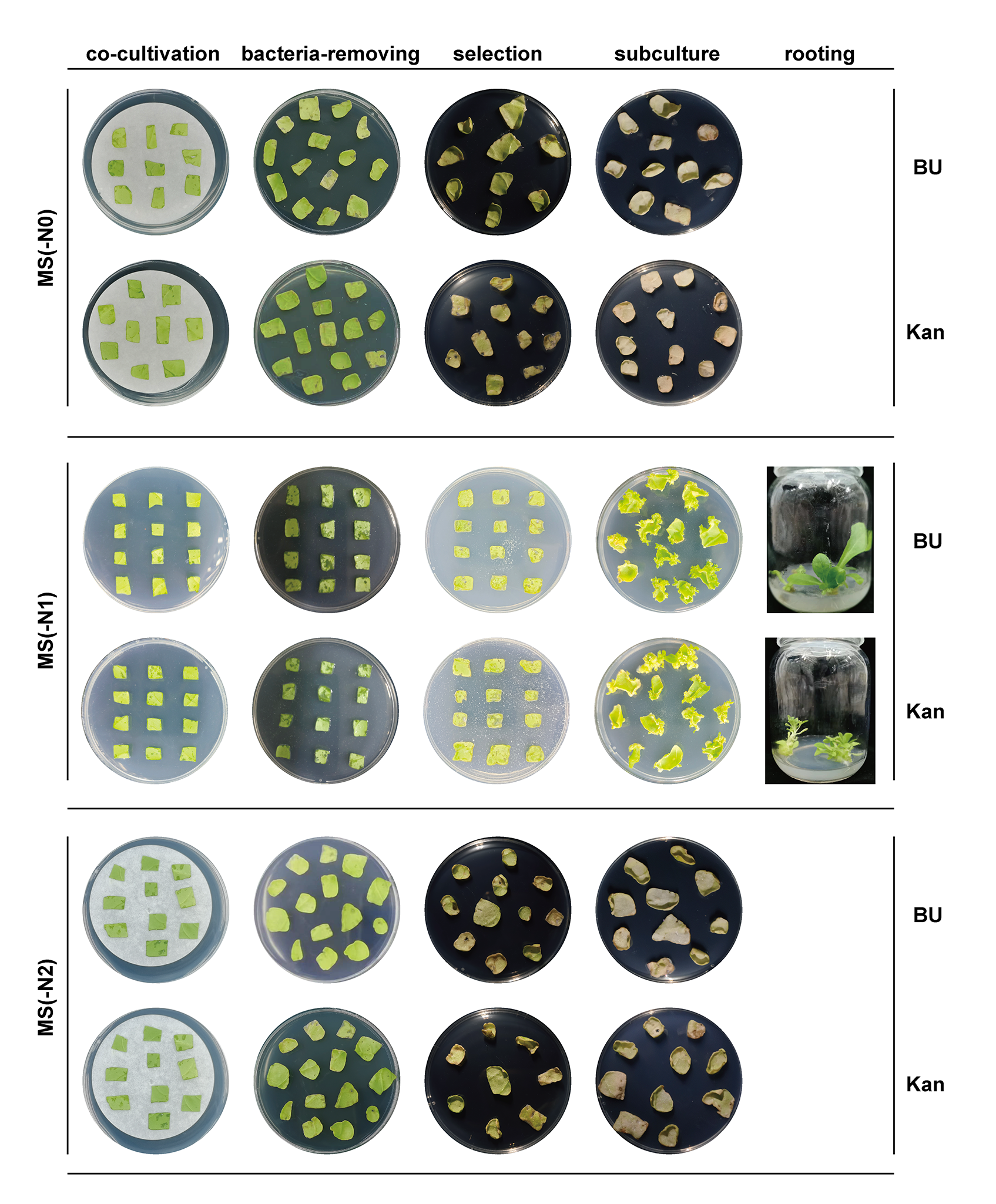

Supplement: S2 Fig — Following Agrobacterium-mediated transformation, explants were cultured sequentially on MS(-N0) media,MS(-N1) media and MS(-N2) media with either 1 mM BU or 0.2 mM Kanamycin. (TIF) [file pone.0347957.s002.tif]

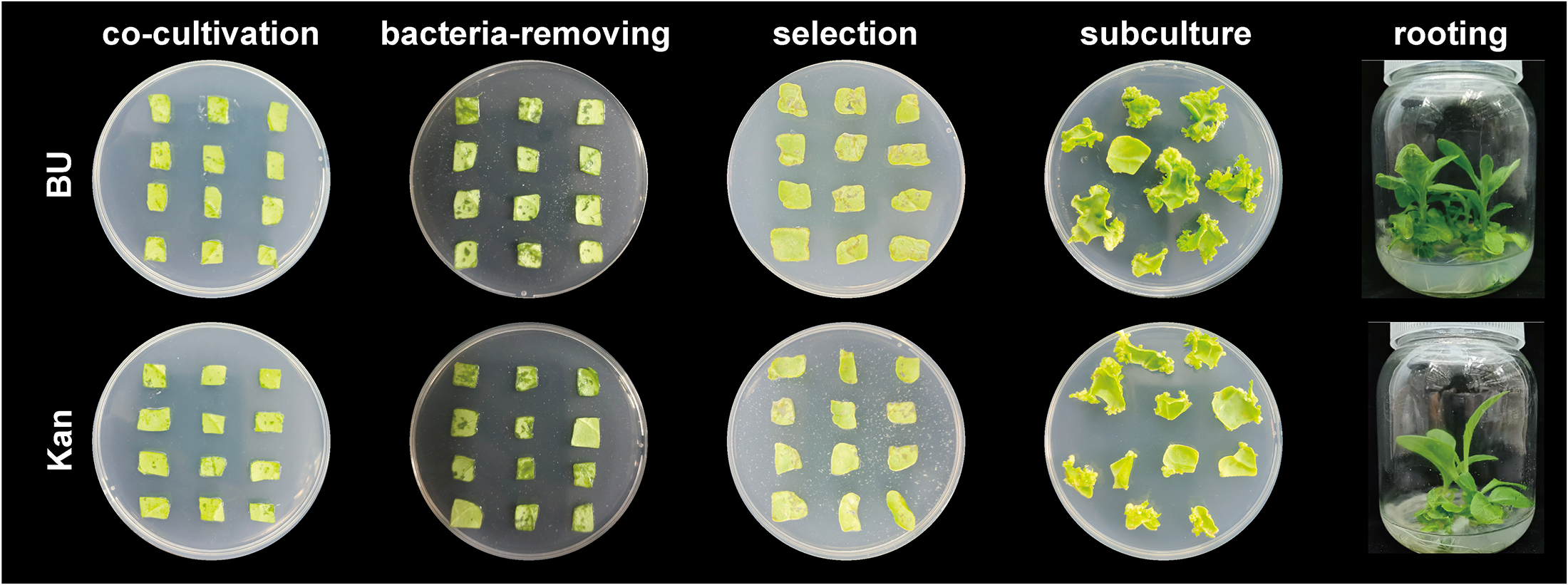

Supplement: S3 Fig — Following Agrobacterium-mediated transformation, explants were cultured sequentially on MS media with either 1 mM BU or 0.2 mM Kanamycin. (TIF) [file pone.0347957.s003.tif]

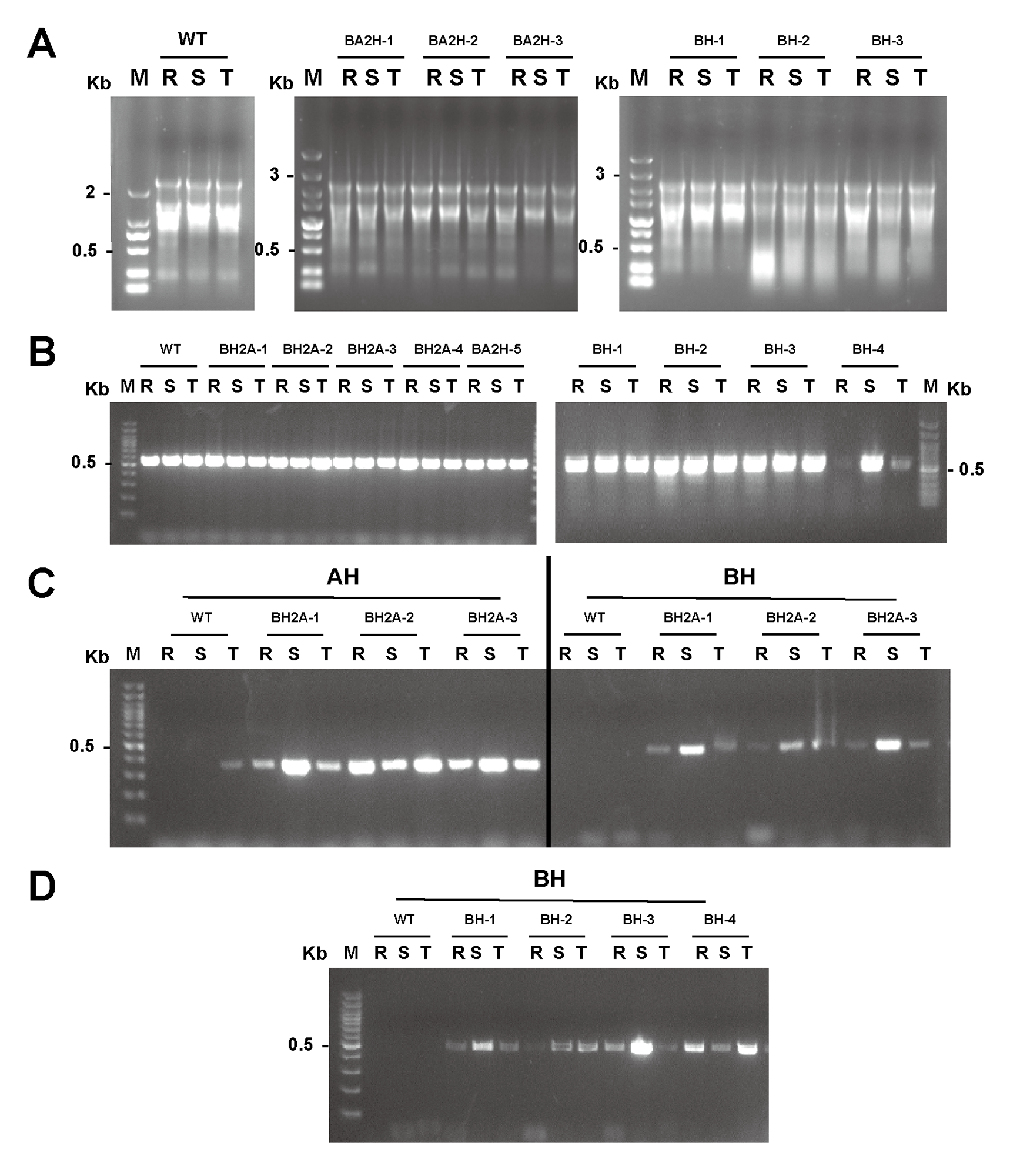

Supplement: S4 Fig — (A) Extracted total RNA; (B) Primer pairs Nt18S-iFw/ Nt18S-iRv were used for RT-PCR of the internal reference gene 18S rRNA to obtain the correct product (552 bp); (C) BA2H transgenic tobacco was amplified using specific primers BH-iFw/ BH-iRv, AH-iFw/ AH-iRv for the BH and AH genes, respectively (AH gene size 373 bp).(D)BH transgenic tobacco amplification using specific primers BH-iFw/ BH-iRv and electrophoretic detection (BH gene size 477 bp). R:root; S:stem; L:leaf; arrows indicate the target PCR bands. (TIF) [file pone.0347957.s004.tif]

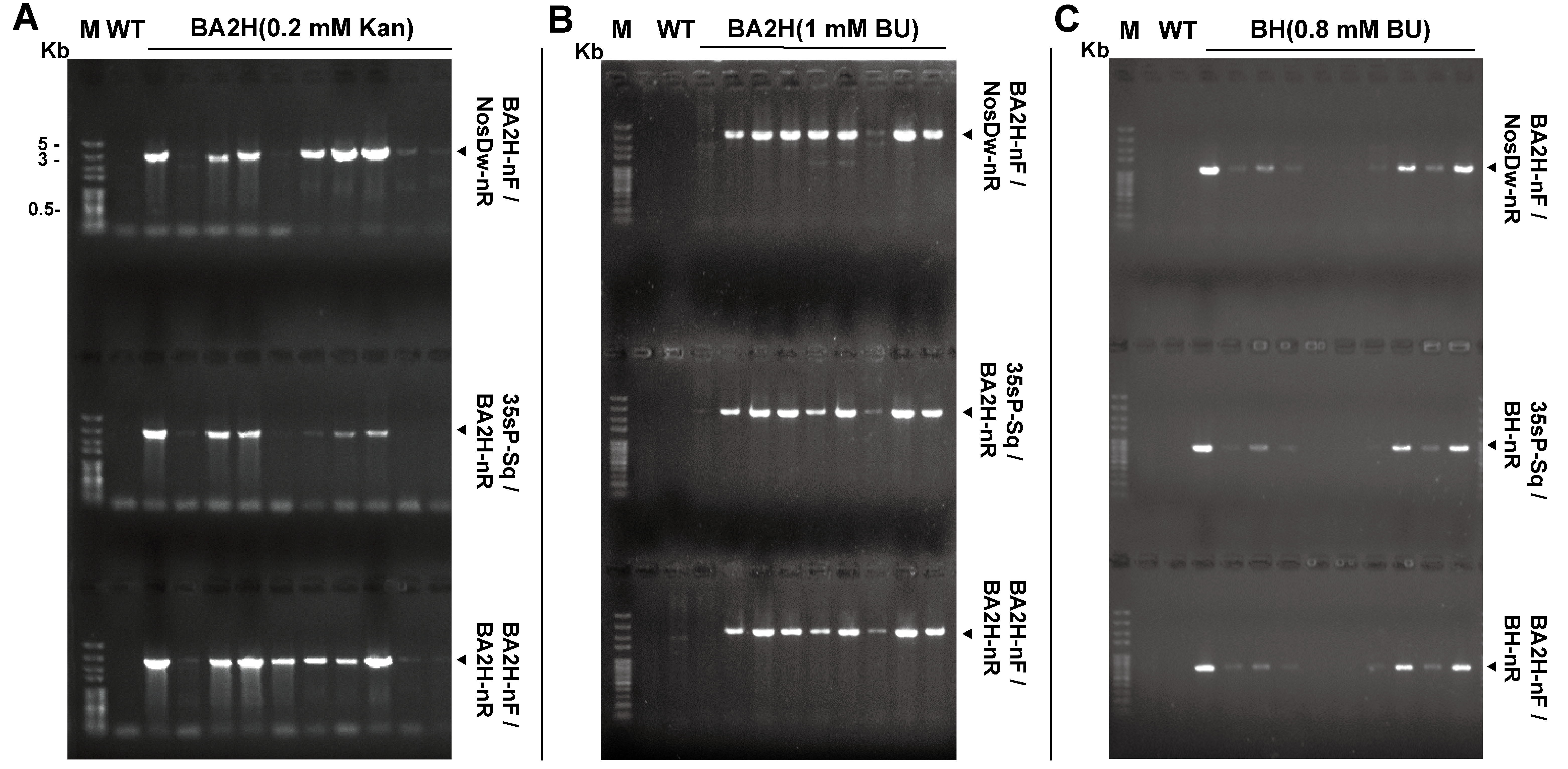

Supplement: S5 Fig — Regenerants obtained under different selection regimes were screened by PCR using three primer sets specific to the transgene. Each lane represents an independent regenerated plantlet. Plants showing amplification with all three primer pairs were considered transgenic-positive. (A) Control: Positive BA2H transgenic plants selected on 0.2 mM kanamycin. (B) BA2H transgenic plants selected on 1 mM BU. (C) BH transgenic plants selected on 0.8 mM BU. (TIF) [file pone.0347957.s005.tif]
