# Supplementary material for: A novel positive selection system for plant transformation based on microbial biuret hydrolase and biuret
Source: PLoS One. 2026 May 8;21(5):e0347957. doi: 10.1371/journal.pone.0347957 (PMC13155557; doi:10.1371/journal.pone.0347957)
Supplement: S1 Table — (DOCX) [file pone.0347957.s006.docx]

****S1 Table . Primers used in this study****

| **Name** | **Sequence (5' to 3')** |
| --- | --- |
| **BA2H-5Nd** | **TAAGAAGGAGATATACATATGGGTCCAGAGTTGTTCATC** |
| **BA2H-3Xh** | **GTGGTGGTGGTGGTGCTCGAGCTTGGCAGCACAGTAAGCAC** |
| **BH-3Xhrc** | **GTGGTGGTGGTGGTGCTCGAGTGCACCAATCACATCTATCAAC** |
| **Tt7Dw-Rv** | **ACCGGATATAGTTCCTCCTTTCAG** |
| **Pt7Up-Fw** | **ACCGCGAAATTAATACGACTCAC** |
| **35sP-Sq** | **GACGCACAATCCCACTATCCTTC** |
| **NosT-Sq** | **GACCGGCAACAGGATTCAATC** |
| **BA2H-5Xb** | **TCCGACTCTAGACACGCTGGAA** |
| **BA2H-3Sc** | **GATTAGAGCTCAGCTCGAGTCACTTGGCAGCAC** |
| **BH-3Xh** | **GCAATTCTCGAGTCATGCACCAATCACATCTATCAAC** |
| **NosDw-nR** | **GTGCTGCAAGGCGATTAAGTTG** |
| **BH-nR** | **GTCATGCACCAATCACATCTATC** |
| **BA2H-nR** | **AGTCACTTGGCAGCACAGTAAG** |
| **BA2H-nF** | **GGGTCCAGAGTTGTTCATCAAG** |
| **Nt18S-iFw** | **GAAACGgCTACCACATCCAAG** |
| **Nt18S-iRv** | **GGCAAATGCTTTCGCAGTTG** |
| **BH-iFw** | **GCCAATACCGCACTCATAGTC** |
| **BH-iRv** | **GGCTTCACGCATTGTAGTATG** |
| **AH-iFw** | **GACCAGATGGACTACCCTTTG** |
| **AH-iRv** | **GCCCAGACTTCTACAGCGAG** |
