# Supplementary material for: A novel positive selection system for plant transformation based on microbial biuret hydrolase and biuret
Source: PLoS One. 2026 May 8;21(5):e0347957. doi: 10.1371/journal.pone.0347957 (PMC13155557; doi:10.1371/journal.pone.0347957)
Supplement: S2 Table — (DOCX) [file pone.0347957.s007.docx]

****S2 Table. Comparison of transformation efficiency and regeneration time between the BU-based and kanamycin-based selection systems.****

| Selection System | BU Concentration (mM) / Kan (mM) | Transformation Efficiency (%) | Regeneration Time (days) |
| --- | --- | --- | --- |
| BH / BU | 0.5 mM BU | 2.2 ± 2.6^c^ | 62 - 70 |
| BH / BU | 0.8 mM BU | 52.6 ± 4.2^b^ | 62 - 70 |
| BH / BU | 1 mM BU | 50.0 ± 5.2^b^ | 62 - 70 |
| BA2H / BU | 0.5 mM BU | 8.7 ± 4.1^c^ | 62 - 70 |
| BA2H / BU | 0.8 mM BU | 13.6 ± 6.4^c^ | 62 - 70 |
| BA2H / BU | 1.0 mM BU | 78.5 ± 5.1^a^ | 62 - 70 |
| NPTII / Kanamycin | 0.2 mM Kan | 66.7 ± 6.3^b^ | 85 - 90 |
| Wild-type negative control | 1.0 mM BU | 0.0 ± 0.0^d^ | - |

*Values are presented as mean ± SD from three independent experiments, each with four technical replicates (20–25 explants per replicate). Different lowercase letters within the Transformation Efficiency column indicate significant differences among treatments at p < 0.05 (one-way ANOVA with Tukey's test). Treatments sharing the same letter are not significantly different. Two controls were included: (i) wild-type explants on 1.0 mM BU showed no regeneration, confirming BU selection efficacy; (ii) wild-type explants on medium without selection showed normal regeneration (91.1%), confirming explant viability under optimal conditions. This growth control was excluded from statistical comparison with selection treatments.*
