# Supplementary material for: A novel positive selection system for plant transformation based on microbial biuret hydrolase and biuret
Source: PLoS One. 2026 May 8;21(5):e0347957. doi: 10.1371/journal.pone.0347957 (PMC13155557; doi:10.1371/journal.pone.0347957)

**The original gel images of S2 Table . Molecular identification of positive transgenic plants under BU-based selection**


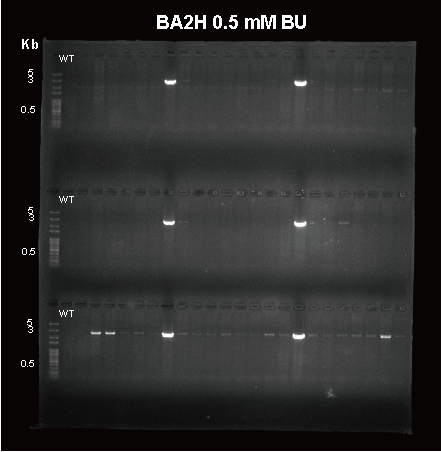


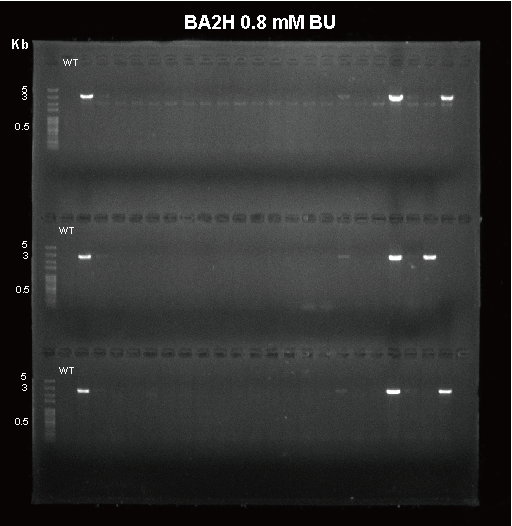

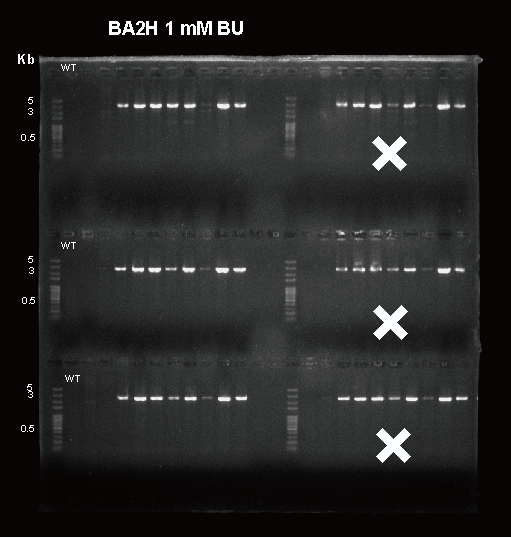

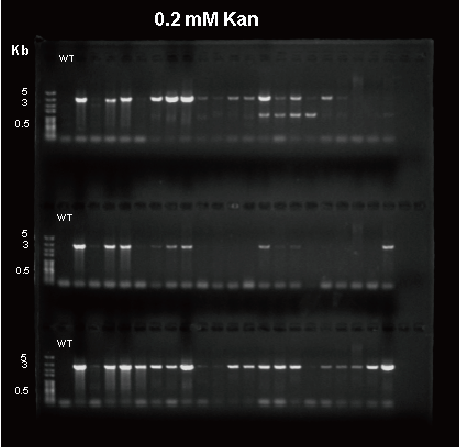

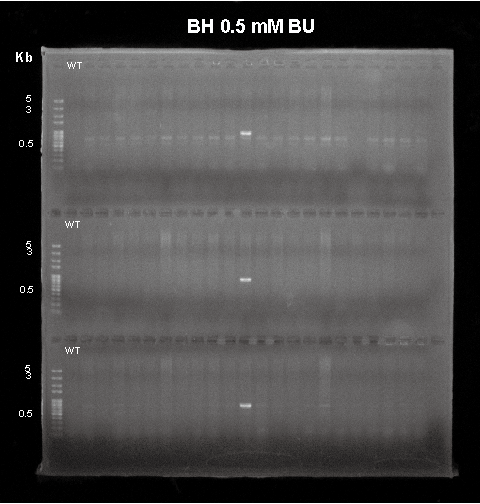

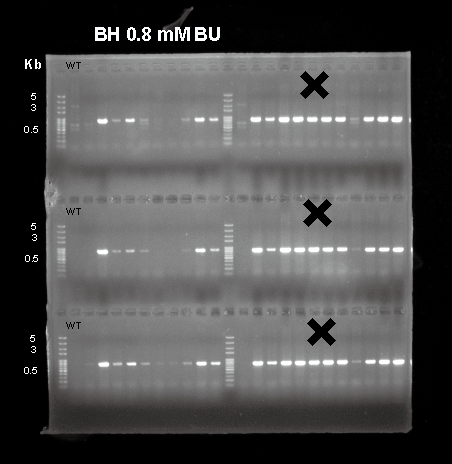

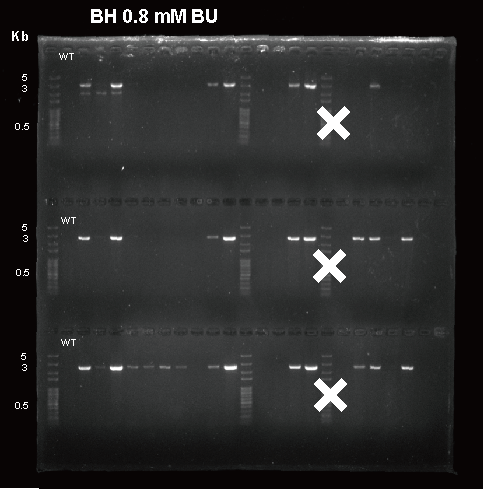


**The original gel images of S4 Fig .RT-PCR analysis of BH and BA2H expression in root, stem, and leaf tissues of BH and BA2H transgenic tobacco**


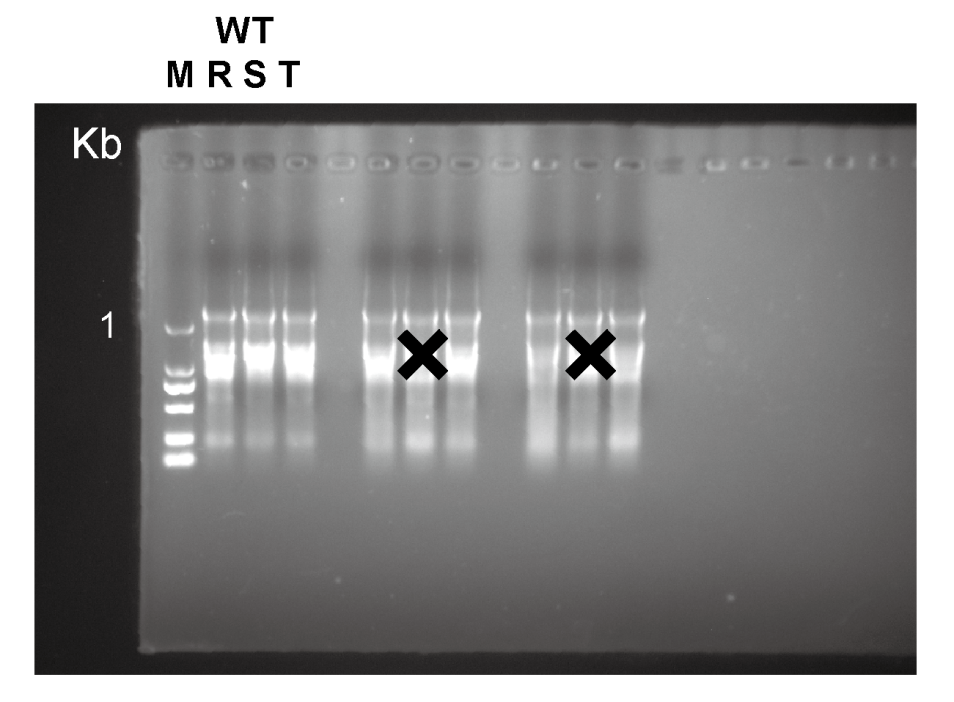


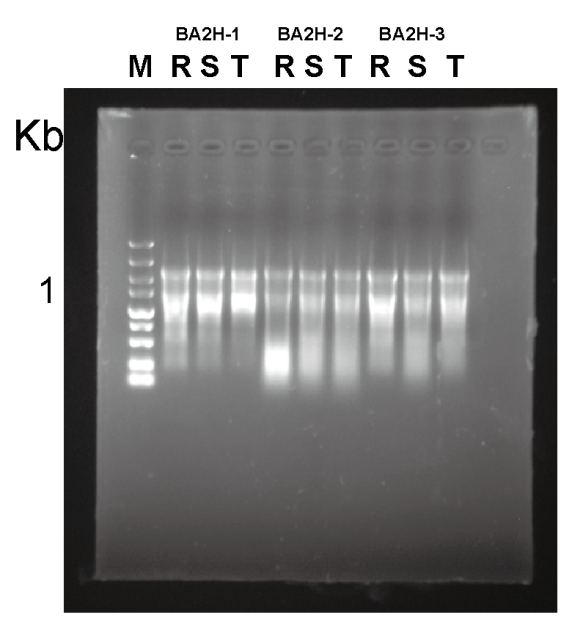

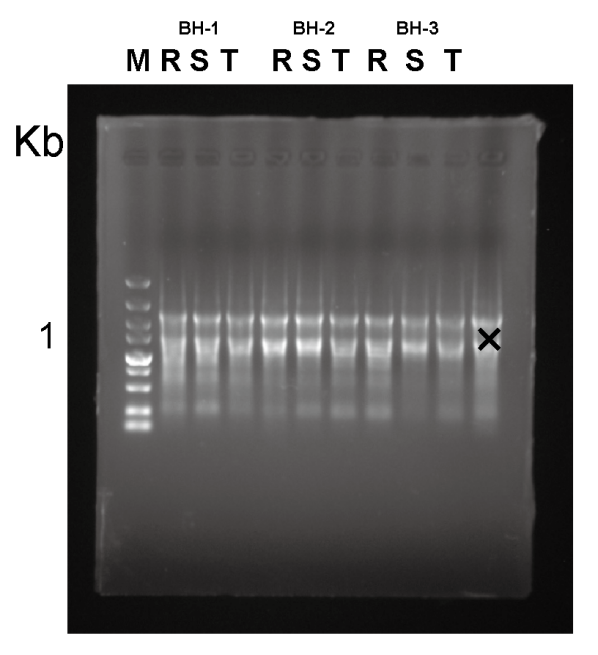

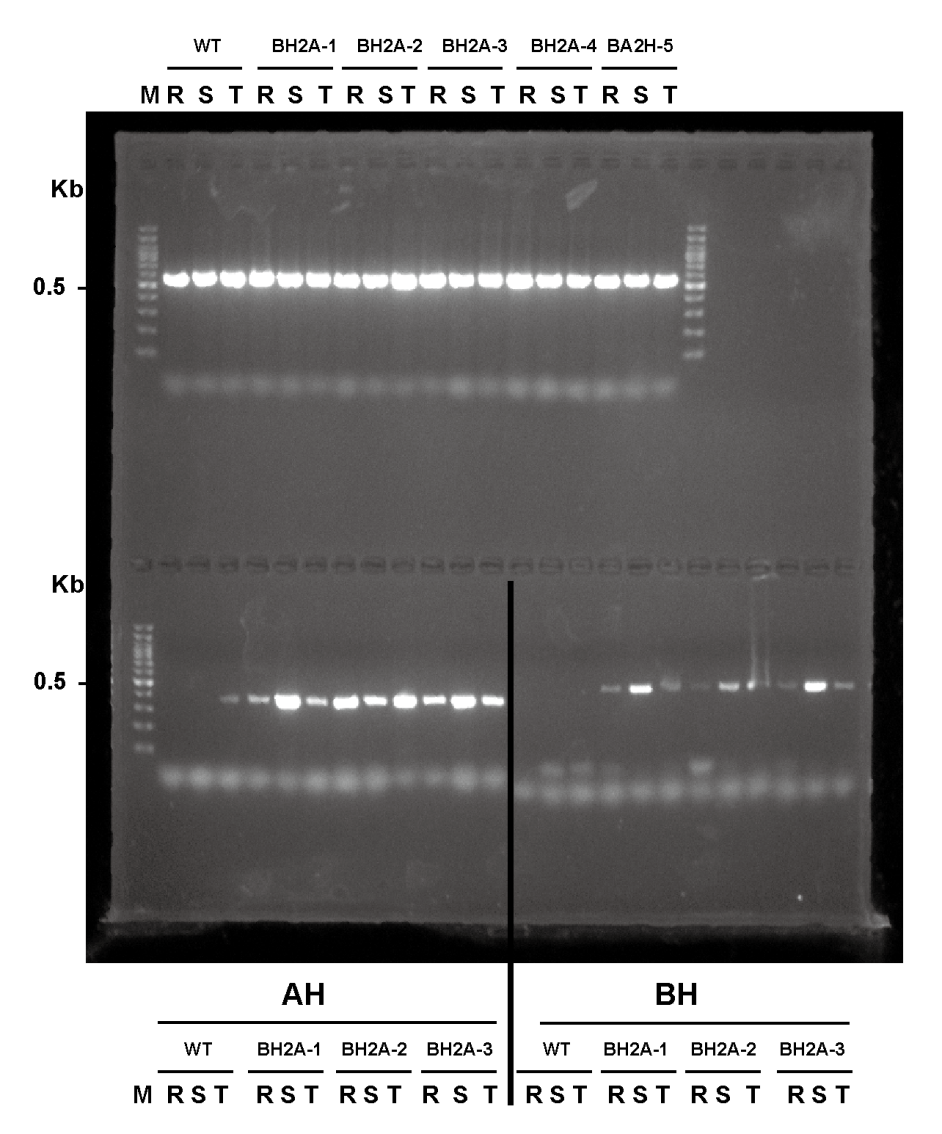


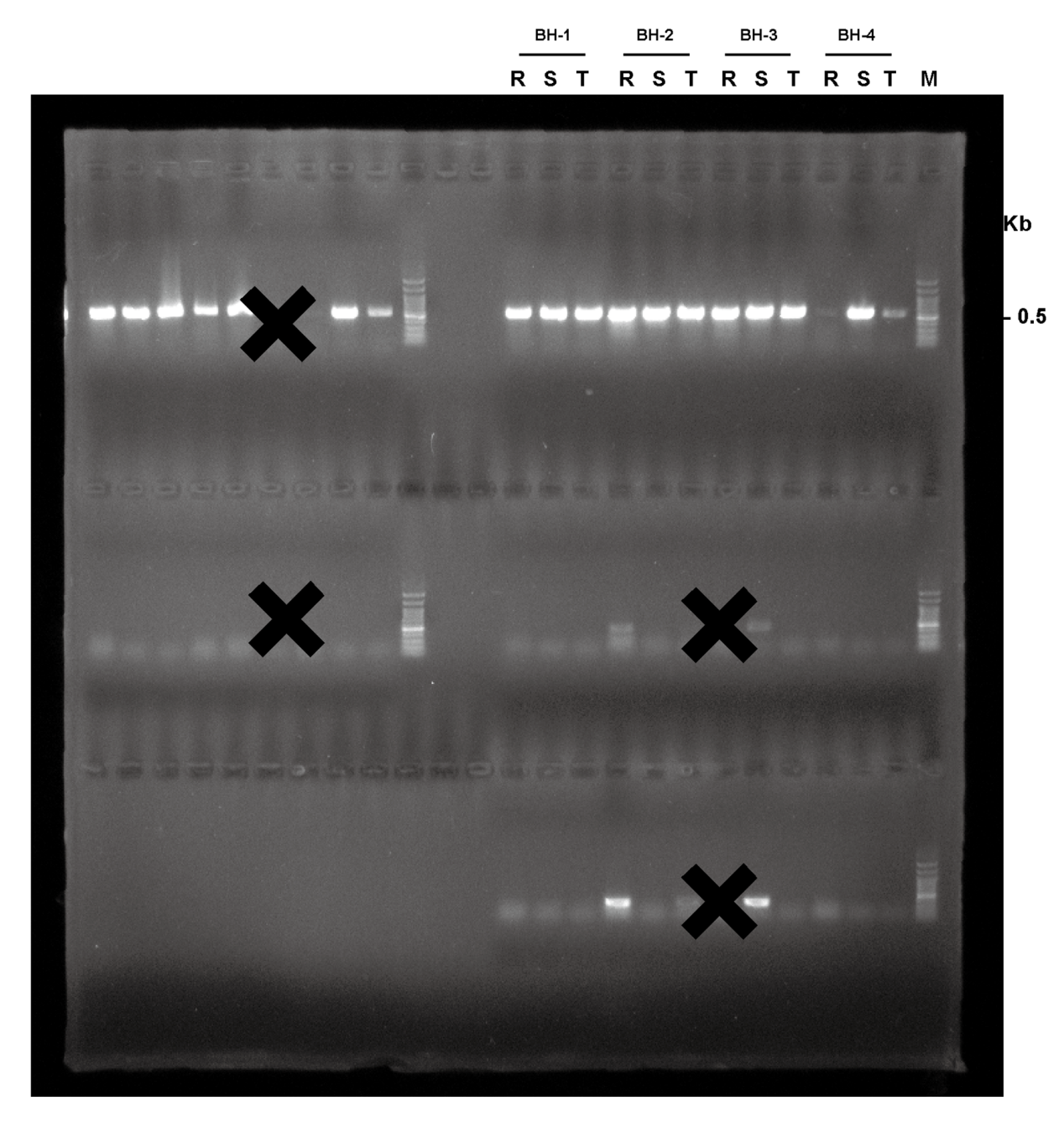

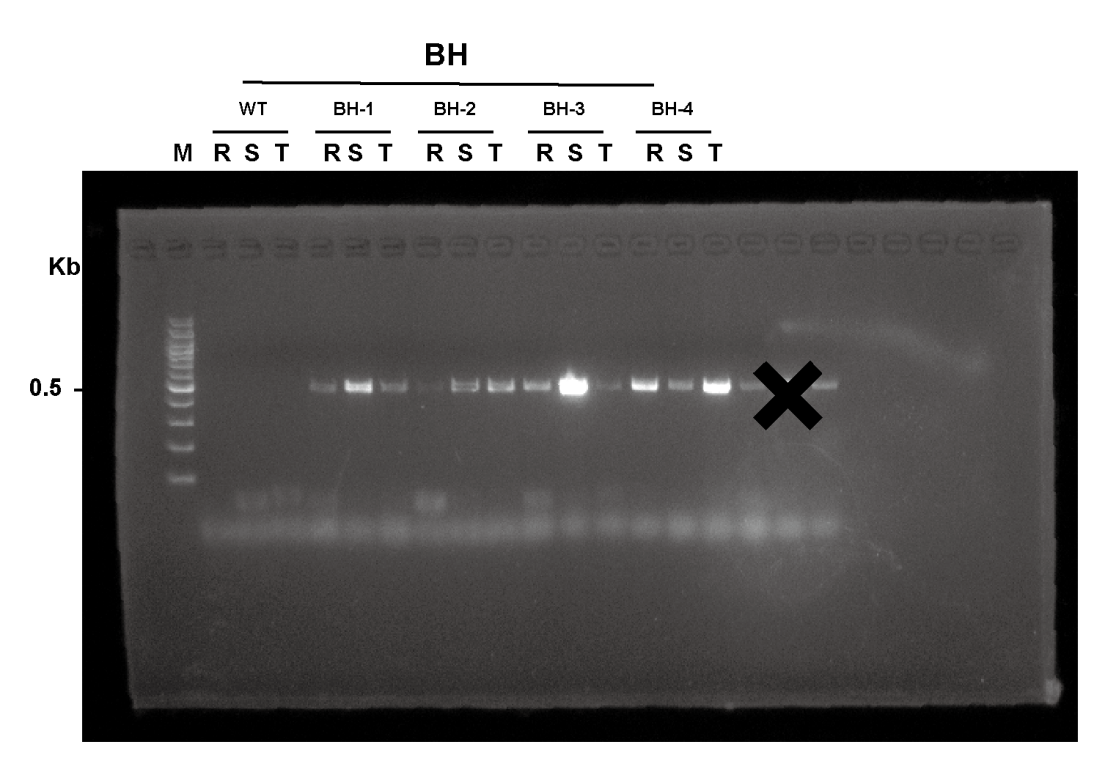

Supplement: S3 File — (DOCX) [file pone.0347957.s008.docx]
