# Supplementary material for: A novel positive selection system for plant transformation based on microbial biuret hydrolase and biuret
Source: PLoS One. 2026 May 8;21(5):e0347957. doi: 10.1371/journal.pone.0347957 (PMC13155557; doi:10.1371/journal.pone.0347957)
Supplement: Attachment — Submitted filename: PONE-D-26-07660 comments.docx [file pone.0347957.s009.docx]

Manuscript No.	PONE-D-26-07660

Manuscript Title:	A Novel Positive Selection System for Plant Transformation Based on Microbial Biuret Hydrolase and Biuret

The study addresses a clearly defined and relevant research problem, providing new evidence that fills an important gap in the existing literature. It uses appropriate and modern approaches to generate findings with practical implications for policy and future research. The topic is timely because it aligns with current priorities and responds to emerging challenges in the field.

1. The experimental design is not described with sufficient clarity to allow replication. For example, the manuscript states that experiments were performed “in triplicate,” but it is unclear whether these are biological replicates (independent experiments) or technical replicates (multiple measurements of the same sample). This distinction directly affects statistical validity.
2. Sample size justification is missing. There is no explanation of how the number of samples or experimental units was determined. For instance, no power analysis or rationale is provided to demonstrate that the study is adequately powered to detect the reported differences.
3. Control treatments are inadequately defined. In some sections, a “control group” is mentioned without clearly specifying its conditions (e.g., untreated, vehicle-treated, or baseline). This makes interpretation of treatment effects ambiguous.
4. Statistical methods are insufficiently justified. The manuscript reports the use of parametric tests (e.g., ANOVA or t-test), but there is no indication that assumptions such as normality or homogeneity of variance were tested. Without this, the validity of the statistical conclusions is uncertain.
5. Exact p-values are not consistently reported. For example, results are described as “significant at p < 0.05” without providing the exact p-value or confidence intervals. This limits transparency and interpretability.
6. Effect sizes are not provided. The manuscript focuses solely on statistical significance without quantifying the magnitude of differences. For instance, reporting a significant increase without indicating percentage change or standardized effect size weakens the biological interpretation.
7. Some conclusions appear to overextend beyond the presented data. For example, the discussion implies a mechanistic explanation for observed results, although no mechanistic experiments were conducted to directly support that claim.
8. Figures lack sufficient clarity. Some graphs do not clearly label units on axes, error bars are not defined (SD vs SE), and figure legends do not adequately explain sample size or statistical comparisons.
9. The data availability statement is absent or incomplete. There is no clear indication of whether raw data, supplementary datasets, or underlying numerical values are accessible in a repository, which is required for transparency.
10. The introduction contains general background information but does not clearly define a specific knowledge gap. For example, while the topic is broadly introduced, the manuscript does not explicitly state what unanswered question this study addresses.
11. The discussion does not critically compare findings with recent literature. Some relevant studies from the past five years are not cited, and where comparisons are made, they are descriptive rather than analytical.
12. Terminology is occasionally imprecise. For example, causal language such as “this treatment induces…” is used even though the study design demonstrates association rather than mechanistic causation.
13. Methodological parameters are incomplete. Critical experimental details such as incubation times, reagent concentrations, environmental conditions, or instrument settings are either briefly mentioned or omitted, which compromises reproducibility.
14. There is limited explanation of variability. In cases where high variance is visible in figures, the manuscript does not discuss possible biological or technical reasons for this dispersion.
15. Language and phrasing occasionally reduce clarity. Some sentences are grammatically awkward or overly long, which makes interpretation of technical details difficult.
