# Supplementary material for: A novel positive selection system for plant transformation based on microbial biuret hydrolase and biuret
Source: PLoS One. 2026 May 8;21(5):e0347957. doi: 10.1371/journal.pone.0347957 (PMC13155557; doi:10.1371/journal.pone.0347957)
Supplement: Attachment — Submitted filename: Response to Reviewers.docx [file pone.0347957.s010.docx]

Dear Editor and Reviewers,

We sincerely thank you for taking the time and effort to review our manuscript (Manuscript ID: PONE-D-26-07660, entitled "A Novel Positive Selection System for Plant Transformation Based on Microbial Biuret Hydrolase and Biuret"). We greatly appreciate your constructive comments and suggestions, which have been invaluable in helping us improve the quality of our work.

We have carefully revised the manuscript according to the editor's and reviewers' comments. Below is our point-by-point response:

****I. Response to the Academic Editor's Comments****

****Response to Editorial Requirements – Copyright Concerns for Figures 2–5****

We thank the editor for raising the copyright concerns regarding Figures 2, 3, 4, and 5. We confirm that ****all of these figures are original works created by the authors specifically for this manuscript****.

****Figure 2**** presents the effect of nitrogen source composition on the sensitivity of wild-type tobacco leaf explants to BU. Explants were cultured for 30 days on media with varying nitrogen compositions (standard MS and MS(−N1)) supplemented with a gradient of BU concentrations (0 to 1 mM). The images and data shown were generated from experiments conducted in our laboratory.

****Figure 3**** documents the regeneration dynamics of BA2H-transgenic tobacco leaf explants under different selection regimes (1 mM BU vs. 0.2 mM kanamycin). The photographs were taken during our transformation experiments and represent original documentation of the regeneration process.

****Figure 4**** shows a comparative analysis of BU tolerance in transgenic and wild-type tobacco seeds. Seeds of WT, BH-transgenic, and BA2H-transgenic lines were germinated on standard MS or nitrogen-deficient media supplemented with 2 mM BU. Panels A–D display germination, shoot growth, and root growth phenotypes, with images 1–3 representing three independent transgenic lines per construct. All images were captured in our laboratory during the germination assays.

****Figure 5**** illustrates the herbicidal activity of BU and the competitive growth advantage of BH-expressing tobacco under BU application. Panel A shows in vitro sensitivity of weed species (Cynodon dactylon and Festuca arundinacea) to BU; Panel B presents a co-culture competition assay with mixed populations of WT tobacco, transgenic tobacco, and weeds; Panel C shows pot-based competition under varying BU concentrations. All data and images were generated from our own greenhouse and laboratory experiments.

As the copyright holders of these original figures, we hereby confirm that they can be published under the CC BY 4.0 license without any restrictions. No permissions from third parties are required.

We have also updated the figure legends to include a brief statement indicating that these are original images created by the authors (e.g., "Original images created by the authors").

Should the editor require any additional documentation or confirmation regarding the originality of these figures, we are happy to provide further details.

****Editor Comment 1: The quality of various figures is low and needs substantial improvement.****

****Response:**** We have recreated all figures to enhance resolution and clarity. Specific improvements include: increased image resolution, optimized layout, and clearer annotations.

****Editor Comment 2: The experimental layout, sample size, treatment plan, control treatments, and replications are not clearly elaborated.****

****Response:**** We have supplemented the "Materials and Methods" section with detailed experimental design information. The revisions can be found on page 4. We have added details regarding the number of independent experiments, replicates per treatment, and control group settings.

****Editor Comment 3: The use of a low concentration (25 μg/mL) of rifampicin alone for Agrobacterium elimination is questionable.****

****Response:**** We thank the editor for highlighting this critical issue. We agree that the original method was inadequate. In the revised manuscript, we have corrected the Agrobacterium elimination method by using 200 mg/L timentin instead. The relevant section has been updated in "Materials and Methods" (page 4).

****Editor Comment 4: The statistical analysis is inadequate.****

****Response:**** We have performed a complete re-analysis of the statistics. This includes testing data for normality and homogeneity of variance, as well as adding significance markers to figures and tables.

****Editor Comment 5: The claims are not justified by the presented data.****

****Response:**** We have re-evaluated the conclusions based on the data and revised them accordingly. Speculative statements lacking direct experimental support have been removed to ensure the conclusions are more rigorous.

****II. Response to Reviewer #1's Comments****

****Reviewer 1-1: The labels in Figure 3 are confusing. Suggest changing them to more descriptive stage names.****

****Response:**** Thank you for the suggestion. We have revised the labels in Figure 3 from "T1-T5" to descriptive stage names: co-cultivation, bacteria-removing, selection, subculture, and rooting. The revised figure can be found on page X.

****Reviewer 1-2: Figure 4 images are too small to see the plants clearly. Wild-type and transgenic plants should be clearly labeled.****

****Response:**** We have recreated Figure 4 with enlarged images and clearly labeled wild-type (WT) and transgenic plants. The revised figure is on pages 9-12, and the figure legend has been updated accordingly.

****Reviewer 1-3: Statistical issues in Table 1 (number of independent experiments, significance markers, inclusion of all treatment data).****

****Response:**** Thank you for your careful review. We have revised Table 1 and added Table S2 in the Supporting Information as follows:

1. The table footnote now clearly states that three independent experiments were performed, each with at least 25 explants.
2. Lowercase letters (a, b, c) have been added to transformation efficiency values to indicate statistically significant differences.
3. All tested treatments are now listed, not just the highest efficiencies.
   The revised Table 1 is on page 9. Statistical significance was determined by Tukey's test (p < 0.05). All data are presented in Table S2.

****Reviewer 1-4: The method for Agrobacterium elimination is incorrect; antibiotics such as carbenicillin should be used instead of rifampicin.****

****Response:**** We are very grateful for pointing out this critical error. We have corrected the method and now use 200 mg/L timentin to eliminate Agrobacterium after co-cultivation. The "Materials and Methods" section (page 4) has been updated accordingly.

****III. Response to Reviewer #2's Comments****

****Reviewer 2-1: The experimental design is unclear, and biological vs. technical replicates are not distinguished.****

****Response:**** Thank you for the suggestion. We have clarified in the "Materials and Methods" section (page 4) that experiments were repeated three times independently (biological replicates), each with four technical replicates. Definitions have been provided.

****Reviewer 2-2: Sample size justification is missing; no rationale for the number of samples is provided.****

****Response:**** We have added a justification for sample size in the "Materials and Methods" section. Based on previous experience from our laboratory [7], we determined that 25–30 explants per treatment would provide sufficient statistical power.

****Reviewer 2-3: Control treatments are inadequately defined.****

****Response:**** We have clearly defined all control groups in the "Materials and Methods" section (page 4): non-transformed wild-type plants as negative control, medium without selective agent as growth control, and kanamycin selection as positive control.

****Reviewer 2-4: Statistical methods are insufficiently justified; normality and homogeneity of variance tests are not mentioned.****

****Response:**** Thank you for this important reminder. We have added a detailed description of the statistical methods in the "Statistical Analysis" subsection (page 4). All data were tested for normality using the Shapiro–Wilk test and for homogeneity of variances using Levene's test before performing ANOVA.

****Reviewer 2-5: Exact p-values are not consistently reported.****

****Response:**** We have revised the Results section to provide exact p-values for all statistical tests (e.g., "p = 0.023") rather than simply stating "p < 0.05."

****Reviewer 2-6: Effect sizes are not provided.****

****Response:**** We have added effect size measures in the Results section. For comparisons of transformation efficiency, we report percentage changes. Details can be found on page 5.

****Reviewer 2-7: Some conclusions overextend beyond the presented data.****

****Response:**** We agree with the reviewer. Speculative mechanistic interpretations lacking direct experimental support have been removed from the Discussion section to ensure all conclusions are appropriately grounded in the data.

****Reviewer 2-8: Figures lack sufficient clarity; axis labels, error bar definitions, etc., are unclear.****

****Response:**** We have recreated all figures. All graphs now clearly label axes and units, figure legends specify whether error bars represent SD or SE, and sample sizes (n) are indicated.

****Reviewer 2-9: The data availability statement is missing or incomplete.****

****Response:**** Thank you for the suggestion. We have added a data availability statement at the end of the manuscript: "All data generated or analyzed during this study are included in this published article and its supplementary information files." (If data are in supplementary materials) or "The datasets generated during the current study are available in the Figshare repository at [URL]." (If uploaded to Figshare). See page X for details.

****Reviewer 2-10: The introduction does not clearly define a specific knowledge gap.****

****Response:**** We have revised the introduction to explicitly state the scientific gap addressed by this study: the low selection efficiency of the Phi/PtxD system in certain crops (e.g., soybean) and the insufficient activity of EcBAP, highlighting the need for a more efficient system. This study shifts the focus from phosphorus-based to nitrogen-based selection, opening a new research direction.

****Reviewer 2-11: The discussion lacks comparison with recent literature; no references from the last five years are cited.****

****Response:**** We have expanded the Discussion section to include comparisons with recent studies published within the last five years. Two new references have been added [17, 18].

****Reviewer 2-12: Terminology is occasionally imprecise, with language implying causal relationships.****

****Response:**** We have carefully reviewed the entire manuscript and revised imprecise terms and language implying causality to more neutral, descriptive phrasing. Changes are distributed throughout the text.

****Reviewer 2-13: Methodological parameters are incomplete, compromising reproducibility.****

****Response:**** We have supplemented the "Materials and Methods" section with all critical experimental parameters. The revised section should now be fully reproducible.

****Reviewer 2-14: There is limited explanation of variability in the data.****

****Response:**** We have added a discussion of potential sources of data variability in the Results section (pages 14-15).

****Reviewer 2-15: Language and phrasing occasionally reduce clarity; some sentences are grammatically awkward or overly long.****

****Response:**** We have polished the language throughout the manuscript, simplifying long sentences and correcting grammatical errors.

Once again, we sincerely thank the editor and reviewers for their valuable comments on our manuscript. These insights have greatly helped us improve the quality of our work. We look forward to your further consideration.

Yours sincerely,

Xiaowei Luo
Corresponding Author
March 18, 2026
